# Pre-frontal parvalbumin interneurons in schizophrenia: a meta-analysis of post-mortem studies

**DOI:** 10.1007/s00702-019-02080-2

**Published:** 2019-09-16

**Authors:** Stephen J. Kaar, Ilinca Angelescu, Tiago Reis Marques, Oliver D. Howes

**Affiliations:** Psychosis Studies [PO63], Inst. of Psychiatry, Psychology and Neuroscience, King’s College London, 5th Floor, Main Building, Denmark Hill, London, UK

**Keywords:** Cognition, Oscillations, Calcium-binding proteins, Neurobiology, Pathology, Immunoreactivity

## Abstract

Parvalbumin interneurons are fast-spiking GABAergic neurons that provide inhibitory control of cortical and subcortical circuits and are thought to be a key locus of the pathophysiology underlying schizophrenia. In view of the contradictory results regarding the nature of parvalbumin post-mortem findings in schizophrenia, we conducted a quantitative meta-analysis of the data on parvalbumin cell density and parvalbumin mRNA levels in pre-frontal regions in the brains of patients with schizophrenia (*n* = 274) compared with healthy controls (*n* = 275). The results suggest that parvalbumin interneurons are reduced in density in the frontal cortex of patients with schizophrenia (*Hedges’ g* = − 0.27; *p* = 0.03) and there is a non-significant reduction in parvalbumin mRNA levels (*g* = − 0.44; *p *= 0.12). However, certain methodological issues need to be considered in interpreting such results and are discussed in more detail. A meta-regression was conducted for post-mortem interval and year of publication as covariates which were both non-significant, except in the mRNA meta-analysis where post-mortem interval was found to be significant. Overall our findings provide tentative support for the hypothesis that the GABAergic system is deficient in schizophrenia and that parvalbumin-containing interneurons offer a potential target for treatment. However, further well-controlled studies that examine multiple regions and layers are warranted to determine whether parvalbumin alterations are region or layer specific and to test the robustness of the findings further.

**Electronic supplementary material:**

The online version of this article (10.1007/s00702-019-02080-2) contains supplementary material, which is available to authorized users.

## Introduction

Schizophrenia is a severe mental disorder characterised by positive, negative and cognitive symptoms (Howes and Murray 2014; Owen et al. 2016). It has ranked 12th in the leading global causes of disability for the last decade (Wang et al. 2016). Current pharmacological treatments, which are all dopamine receptor ligands (Howes et al. 2009), are ineffective for many patients, highlighting the need for a better understanding of the neurobiology underlying the disorder to develop more effective treatments (Howes and Kapur 2014; Nutt and Need 2014). A number of neurotransmitters, including dopamine, glutamate and gamma-aminobutyric acid (GABA), are implicated in the pathophysiology of schizophrenia (Frohlich and Van Horn 2014; Howes et al. 2012; Krystal et al. 1994). GABAergic interneurons provide inhibitory control of cortical and subcortical circuits (Curley and Lewis 2012; Konradi et al. 2011) and are, therefore, thought to lead to glutamatergic and dopaminergic dysfunction, which in turn leads to the symptoms of schizophrenia (Grace 2016). Abnormal GABAergic interneuron activity is thought to play a key role in working memory and other cognitive symptoms of schizophrenia (Frankle et al. 2015; Gonzalez-Burgos et al. 2015; Heckers and Konradi 2015; Lewis et al. 2012; Perry et al. 1979; Stedehouder and Kushner 2016; Taylor and Tso 2015).

Although more than 20 different classes of GABAergic interneurons exist (DeFelipe et al. 2013), it has been suggested that in schizophrenia there is a loss of a particular type of GABAergic interneurons characterised by containing the calcium-binding protein parvalbumin (PV) (Beasley and Reynolds 1997; Chung et al. 2016a; Enwright Iii et al. 2017; Lisman et al. 2008; Lodge and Grace 2008; Perez and Lodge 2013). Parvalbumin interneurons are fast-spiking neurons that are critical to the generation of gamma oscillations, a type of high-frequency neuronal oscillation linked to working memory and other cognitive processes in healthy subjects and patients with schizophrenia (Gonzalez-Burgos et al. 2015; Sohal et al. 2009; Tallon-Baudry et al. 1998; Uhlhaas and Singer 2010). So far, several post-mortem studies have examined parvalbumin interneurons in patients with schizophrenia, though the findings have not all been consistent (Chung et al. 2016a; Gonzalez-Burgos et al. 2015) with some early studies showing decreases in parvalbumin interneuron density in schizophrenia (Beasley and Reynolds 1997; Reynolds et al. 2002) whilst others failed to find significant differences between patients and controls (Enwright et al. 2016; Hashimoto et al. 2003; Woo et al. 1997).

Contemporary interpretations of the literature highlight the methodological limitations in measuring parvalbumin cell density; in particular, the danger of using immunoreactivity labelling techniques when protein expression is decreased (Enwright Iii et al. 2017). The prevailing view, therefore, remains that in schizophrenia parvalbumin interneuron abnormalities exist not at the level of neuronal morphology or density, but at the molecular level of gene expression and protein synthesis (Chung et al. 2016a; Enwright Iii et al. 2017; Enwright et al. 2016; Fung et al. 2014; Hashimoto et al. 2008; Joshi et al. 2015; Volk et al. 2016a). However, Toker et al. (2018) have challenged this view in a large transcriptomic study on bulk post-mortem tissue, using cell type-specific marker genes to indirectly show reduced parvalbumin cell density in schizophrenia. As far as the authors are aware, there has not been a previous meta-analysis of parvalbumin post-mortem studies in the pre-frontal cortex. In view of this, we aimed to systematically review and meta-analyse the available evidence on parvalbumin neuronal density and mRNA brain measures in the pre-frontal cortex in people with schizophrenia.

## Methods and materials

### Search strategy

A standardised search was conducted according to the PRISMA guidelines (Liberati et al. 2009). The Medline, EMBASE and PsycINFO electronic databases were searched. The electronic search using EMBASE and PsycINFO was carried out together using Ovid. The following keywords were used “(schizophrenia OR psychotic disorders OR psychosis) AND (parvalbumin)” until 8th February 2019 (for details of search strategy, see supplementary information).

### Study selection

Inclusion criteria were (1) original studies reporting post-mortem findings of parvalbumin neuronal density or parvalbumin mRNA levels in the pre-frontal cortex (2) included patients with a confirmed diagnosis of schizophrenia (including schizoaffective disorder) (3) reporting mean and variance measures for patient and control groups. Exclusion criteria were pre-natal studies, animal studies, and articles in a language other than English, review articles not reporting original data, single cases reports, studies without a control group and articles that were not published in peer-reviewed journals. Studies that only measured GABA-related transcripts or markers but not parvalbumin protein or mRNA were excluded. We did not include studies that did not measure parvalbumin cell density and/or measured mRNA in regions other than the pre-frontal cortex, e.g. Konradi et al. (2011). We also did not include micro-array studies looking at pre-frontal regions (*n* = 4) (Catts and Weickert 2012; Enwright Iii et al. 2017; Fung et al. 2010; Mellios et al. 2009) as following exclusion for sample overlap or reuse of data, there were insufficient studies (*n* = 2) (Fung et al. 2010; Mellios et al. 2009) for a quantitative meta-analysis; however, the results of these studies are discussed later in the paper.

### Data extraction

The study has two outcome measures: (1) the effect size for the difference in parvalbumin neuronal density between patients and controls, i.e. the density of cells defined by a presence or absence of detectable levels of parvalbumin protein or parvalbumin mRNA [either in neuron/mm^2^ or neuron/mm^3^ or neurons per identical volumes of sampled tissue in the case of Chung et al. (2016a)]; 2) the effect size for the difference parvalbumin mRNA levels per neuron or in total grey matter between patients and controls. For all studies we extracted the mean and standard deviation for neuronal density and parvalbumin mRNA for each pre-frontal region in patients with schizophrenia and healthy controls using the available published data. WebPlotDigitizer (https://automeris.io/WebPlotDigitizer) was used to extract data from plots when necessary. If cell density or mRNA was measured across different laminar layers, these data were combined into one simple mean for the region. The online software package StatsToDo (StatsToDo 2014) was used to combine *n*, standard deviation (sd) and means for each laminar layer for each region into one overall *n*, mean and sd for each group (patient or control) in each region. In addition, we extracted the following variables: sample size, mean age, mean post-mortem interval, psychiatric medication, mode of death and methodology of the study. We took particular care to ensure studies which shared brain collections did not include the same brains and, in those studies, included in the quantitative meta-analysis that shared the same collection contact was made with the lead author to confirm no overlap. This led to the exclusion of a number of studies with overlapping brains (Beasley et al. 2002; Bitanihirwe et al. 2009; Catts and Weickert 2012; Chung et al. 2016b; Enwright Iii et al. 2017; Hashimoto et al. 2005, 2008; Knable et al. 2004; Lewis 2000; Torrey et al. 2005). In the case of the Hashimoto et al. (2003) and Volk et al. (2016a) studies, means and standard deviations were calculated using raw data from the latter study that excluded the overlapping subjects. All studies included in the quantitative meta-analysis excluded patients with a history of or post-mortem findings suggestive of a neurological or neurodegenerative disease.

### Data analysis

RStudio statistical software running the “metafor” package was used to perform the meta-analysis (RStudio Team 2015). Group size, mean and standard deviation were used to determine standardised mean effect size (*Hedges’ g*). A random-effects meta-analytic model was used that did not assume homogeneity of effects. Heterogeneity was measured by calculating *I*^2^. Meta-regression was used to assess the effects of year of publication and PMI. Publication bias was tested for using funnel plot asymmetry and regression test. Where potential publication bias was suspected, trim and fill analysis was conducted to correct for putatively missing studies. A *p* value < 0.05 (two tailed) was taken as a significance level. Sub-analyses by fixation method (paraformaldehyde or formalin/paraffin) and by laminar layers III and IV were performed on the pre-frontal cortex cell density data (see Figs. 3 and 4).

## Results

**Fig. 1:**
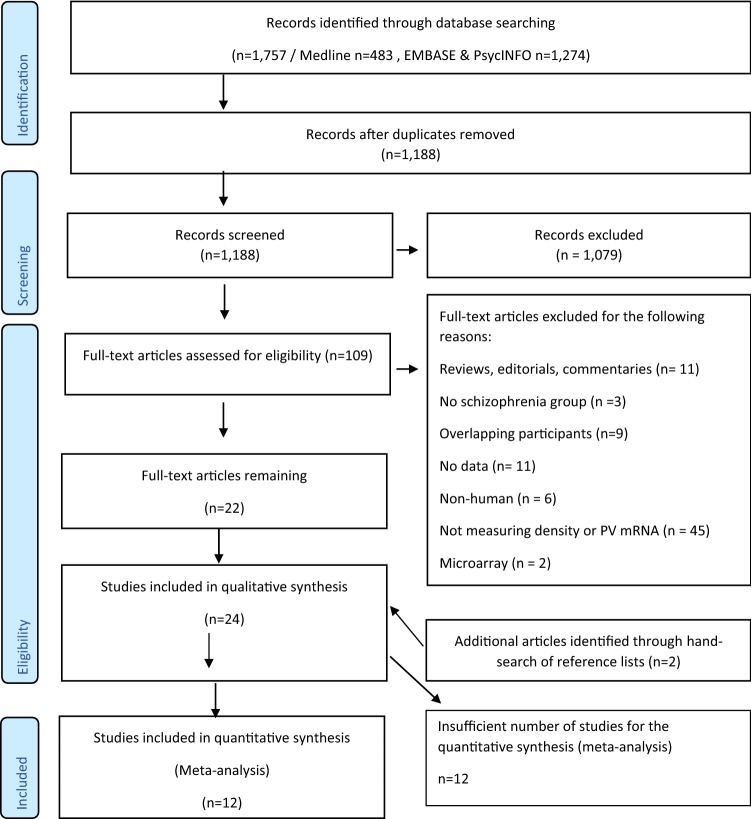
Flowchart showing the inclusion of studies for the meta-analysis

The searches identified 1188 publications, once duplicates were removed (see Fig. 1 for a PRISMA diagram of the literature search). Following screening, 24 post-mortem studies were identified (see Table 1 Supplementary Information for study characteristics). In total, 12 studies were conducted in pre-frontal regions, so were taken forward to the meta-analysis. Tables 1 and 2 summarise the clinico-demographic characteristics of the subjects included as well as the methodological approaches used, in the meta-analyses of parvalbumin cell density (*n* = 9) and parvalbumin mRNA (*n* = 4), respectively. This final sample (*n* = 13—Hashimoto et al. (2003) presents data for both parvalbumin cell density and mRNA measures) included 274 patients with schizophrenia (mean age: 49.8) and 275 healthy controls (mean age: 50.6).

**Table 1:** Characteristics of studies included in parvalbumin cell density meta-analysis

Author and year	Outcome measure included in meta-analysis	Schizophrenia (SCZ) patients/controls (CON) (*n*)	Age mean (sd) (years): schizophrenia (SCZ)/controls (CON)	Method	Area	Cause of death	Patients’ medication	Mean (sd) post-mortem interval (hr): schizophrenia (SCZ) patients/controls (CON)	Significant findings (*p* < 0.05) (schizophrenia relative to controls) (summary)
Woo et al. (1997)	Parvalbumin (PV) cell density (neurons/mm^2^)	15/15	53.6 (± 13)/53.9 (± 13.8)	Immunocytochemistry (ICC)	Pre-frontal cortex (BA 9 and 46)	SCZ: 5 suicide CON: 0 suicide	SCZ: 8 or more treated with antipsychotics CON: x1 antipsychotics	11.7 (± 5.6)/11.3(± 5.4)	↔ Parvalbumin (PV) interneuron density in pre-frontal cortex
Beasley and Reynolds (1997)	PV cell density (neurons/mm^2^)	18/22	69.5 (± 3.7)/62.7 (± 4.0)	ICC	Pre-frontal (BA 10)	SCZ: unknown CON: all sudden death	Unavailable	33.8 (±10.9/42.3(± 4.1)	↓ PV interneuron density in frontal cortex
Reynolds et al. (2002)	PV cell density (neurons/mm^2^)	15/15	44.2 (range 25–62)/48.1 (range 29–68)	ICC	Dorsolateral pre-frontal cortex (BA 46)	SCZ: 4 suicide CON: 0 suicide	SCZ: 14/15 recieved antipsychotics	33.7 (range 12–61)/23.7 (range 8–42)	↓ PV neuron density in DLPFC and EC
Hashimoto et al. (2003)	PV cell density (neurons/mm^2^)	15/15	43.0 (± 12)/43.3 (± 14.6)	In situ hybridisation (ISH) for parvalbumin	Pre-frontal cortical region (BA 9)	SCZ: 4 suicide CON: 0 suicide	3 SCZ unmedicated (antipsychotic) at death	16.9 (± 8.0)/17.0 (± 5.8)	↔ PV interneuron density in PFC ↓ PV interneuron mRNA in PFC
Tooney and Chahl (2004)	PV cell density (neurons/mm^2^)	6/6	44 (± 16)/43 (± 14)	ICC	Pre-frontal cortical region (BA 9)	SCZ: 2 suicide CON: 3 suicide	All SCZ had taken various antipsychotic medications	16.9 ((± 9.5)/20.6 ((± 7.0)	↔ PV interneuron density in PFC
Sakai et al. (2008)	PV cell density (neurons/mm^2^)	7/5	47.4 (± 7.63)/56.8 (± 5.81)	ICC	Pre-frontal cortex (BA 9)	SCZ: 2 cardiorespiratory failure, 1 renal failure, 3 cancer, 1 thyroid crisis CON: 3 cardiorespiratory failure, 2 liver failure	Not available	Not available	↓ PV interneuron density in PFC layers 4
Bitanihirwe and Woo (2014)	Density of cells positive for both PV and GAT-1 (neurons/mm^2^)	20/20	60.2 (± 16.7)/60.4 (± 17.3)	Dual ISH for parvalbumin and GABA transporter GAT-1	Pre-frontal cortex (BA 9)	SCZ: 2 suicide	SCZ - 17 multiple psychotropics	19.8 (± 0.28)/18.7 (± 5.5)	↓ GAT-1 + PV interneuron density
Chung et al. (2016a)	PV cell density (neurons/identical volume)	20/20	45.2 (± 11.8)/46.3 (± 12.1)	ICC	Dorsolateral pre-frontal (layer 4) (BA 9)	SCZ: 8 suicide	SCZ 19/20 antipsychotics	15.4 (± 6.3)/16.4 (± 5.5)	↔ PV neuron density in layer 4 ↓ density of VGlut1 +/PSD95 + puncta on PV interneurons
Enwright et al. (2016)	PV cell density (neurons/m^3^)	20/20	45.8 (± 9.5)/47 (± 10.1)	ICC	Dorsolateral pre-frontal (BA 9)	SCZ: 3 suicide	SCZ 17/20 antipsychotics	13.2 (± 7.7/)13.1 (± 6.2)	↔ Densities of PV cells and of PNNs in DLPFC ↓ PV immunoreactivity in cell bodies and in individual PNNs around PV cells

↔ = No significant difference between patients and controls, ↓ = significant reduction in patients

**Table 2:** Characteristics of studies included in parvalbumin mRNA meta-analysis

Author and year	Outcome measure included in the meta-analysis	Schizophrenia (SCZ) patients/controls (CON) (*n*)	Age mean (sd) (years): schizophrenia (SCZ)/controls (CON)	Method	Area	Cause of death	Patients’ medication	Mean (sd) post-mortem interval (h): schizophrenia (SCZ) patients/controls (CON)	Significant findings (*p* < 0.05) (schizophrenia relative to controls) (summary)
Hashimoto et al. (2003)	Parvalbumin mRNA per neuron	15/15	43.0 (± 12)/43.3 (± 14.6)	In situ (ISH) film radiography	Pre-frontal cortical region (BA 9)	SCZ: 4 suicide CON: 0 suicide	3 SCZ unmedicated (antipsychotic) at death	16.9 (± 8.0)/17.0 (± 5.8)	↔ PV interneuron density in PFC ↓ PV interneuron mRNA in PFC
Fung et al. (2014)	Parvalbumin mRNA in total grey matter	35/34	42.6 (19–59)/43.8 (31–60)	qPCR	Dorsolateral pre-frontal cortex (BA 46) and lateral orbitofrontal cortex	SCZ: 7 suicide	85004.3 (100335) (lifetime fluphenazine mg equiv.)	31.4 (15.4)/29.5 (13.0)	↔ PV mRNA in DLPFC or lateral OFC
Joshi et al. (2015)	PV mRNA expression in grey matter	38/38	52.24 (± 14.52)/52.55(± 14.51)	qPCR	Orbital frontal cortex	Not available	SCZ 35/38 antipsychotics, 3 unknown; SCZ 19/38 on antidepressants, 3 uknown	28.21(± 13.57)/26.43(± 11.69)	↓ PV mRNA in OFC (↔ using qPCR)
Volk et al. (2016a)	PV mRNA levels in grey matter	50/50	48.62(± 12.7)/50.06 (± 13.2)	qPCR	Pre-frontal cortex (BA 9)	SCZ: 10 suicide	89.7% SCZ on antipsychotics at time of death	21.07 (± 11.9)/20.8(± 11.5)	↓ PV mRNA in “Low LGM” group ↔ PV mRNA in “non-LGM” group

### Cell density

**Fig. 2:**
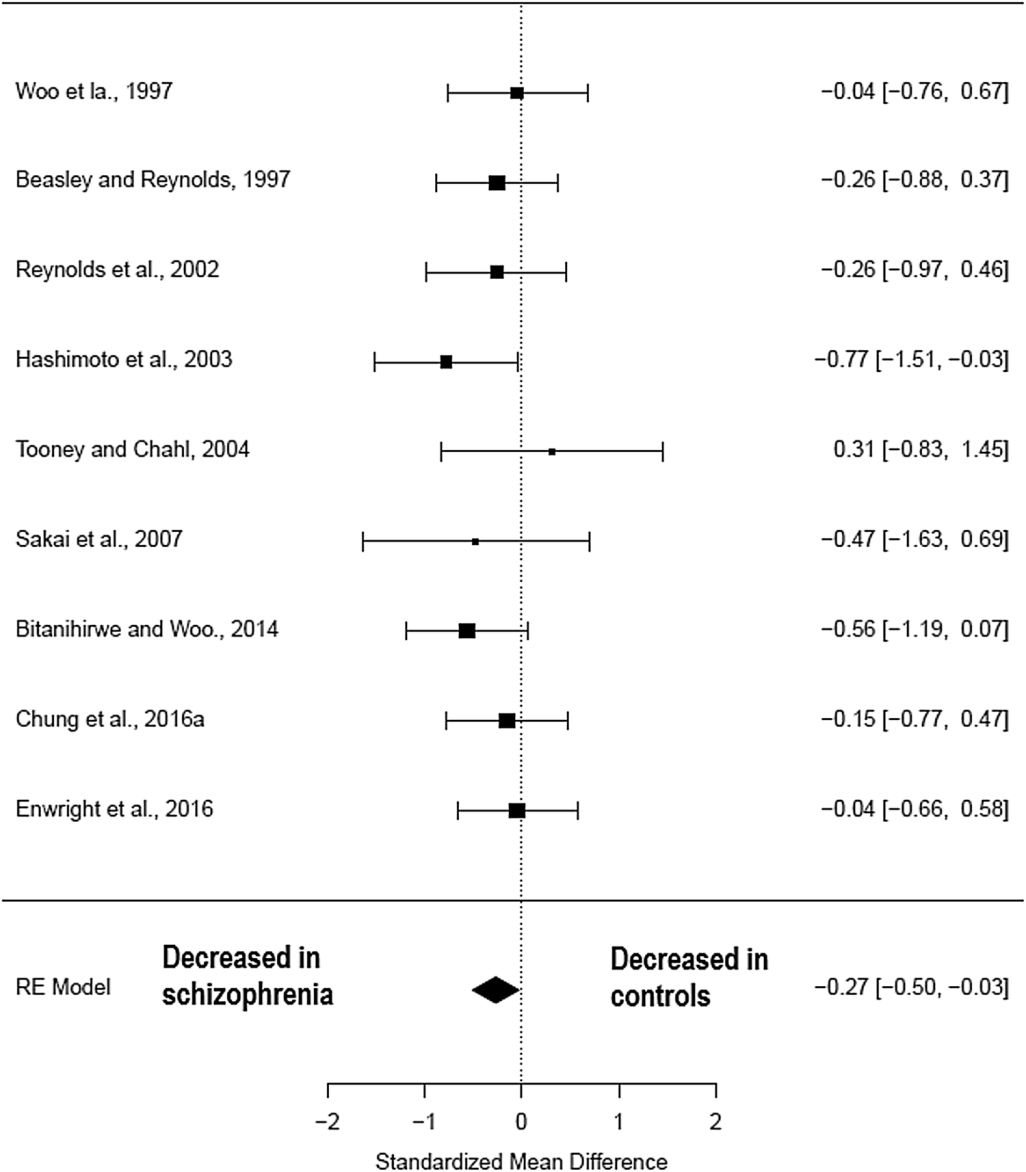
Meta-analysis of studies measuring parvalbumin neuron density in the pre-frontal cortex

Nine studies (comprising 136 schizophrenia patients and 138 healthy controls) (Table 1) measured parvalbumin neuron density in the pre-frontal cortex. There was a significant reduction in parvalbumin cell density in the pre-frontal cortex regions of patients with schizophrenia relative to healthy controls (Hedges’ *g* = − 0.27; *z* = − 2.17; *p* = 0.03; 95% confidence interval (CI): − 0.51 to − 0.03) (Fig. 2). The *I*^2^ test revealed low heterogeneity (*I*^2^ = 0%; 95% CI: 0–57.53%). Visual inspection of the funnel plot analysis revealed one possible missing study on the left side (Supplementary Fig. 1). The funnel plot regression test was not significant (*t* = 0.28, *df* = 7, *p* = 0.8). After correction for potential publication bias the effect size reduction seen in parvalbumin cell density in patients with schizophrenia remained significant (Hedges’ *g* = − 0.29; *p* = 0.01). A sub-analysis by fixation method of the parvalbumin cell density studies in the pre-frontal cortex showed that those studies using paraformaldehyde, a more reliable fixation method, showed a non-significant reduction in patients with schizophrenia compared with healthy controls (Hedges’ *g* − 0.30; *p* = 0.05; CI: − 0.59–0.02) as did the formalin/paraffin-prepared studies (Hedges’ *g* − 0.19; *p *= 0.35; CI: − 0.60–0.22) (see Fig. 4). Leave-one-out analysis revealed that the results became non-significant if the Hashimoto et al. (2003) or the Bitanihirwe and Woo (2014) studies were taken out (see Supplementary Information).

### mRNA

**Fig. 3:**
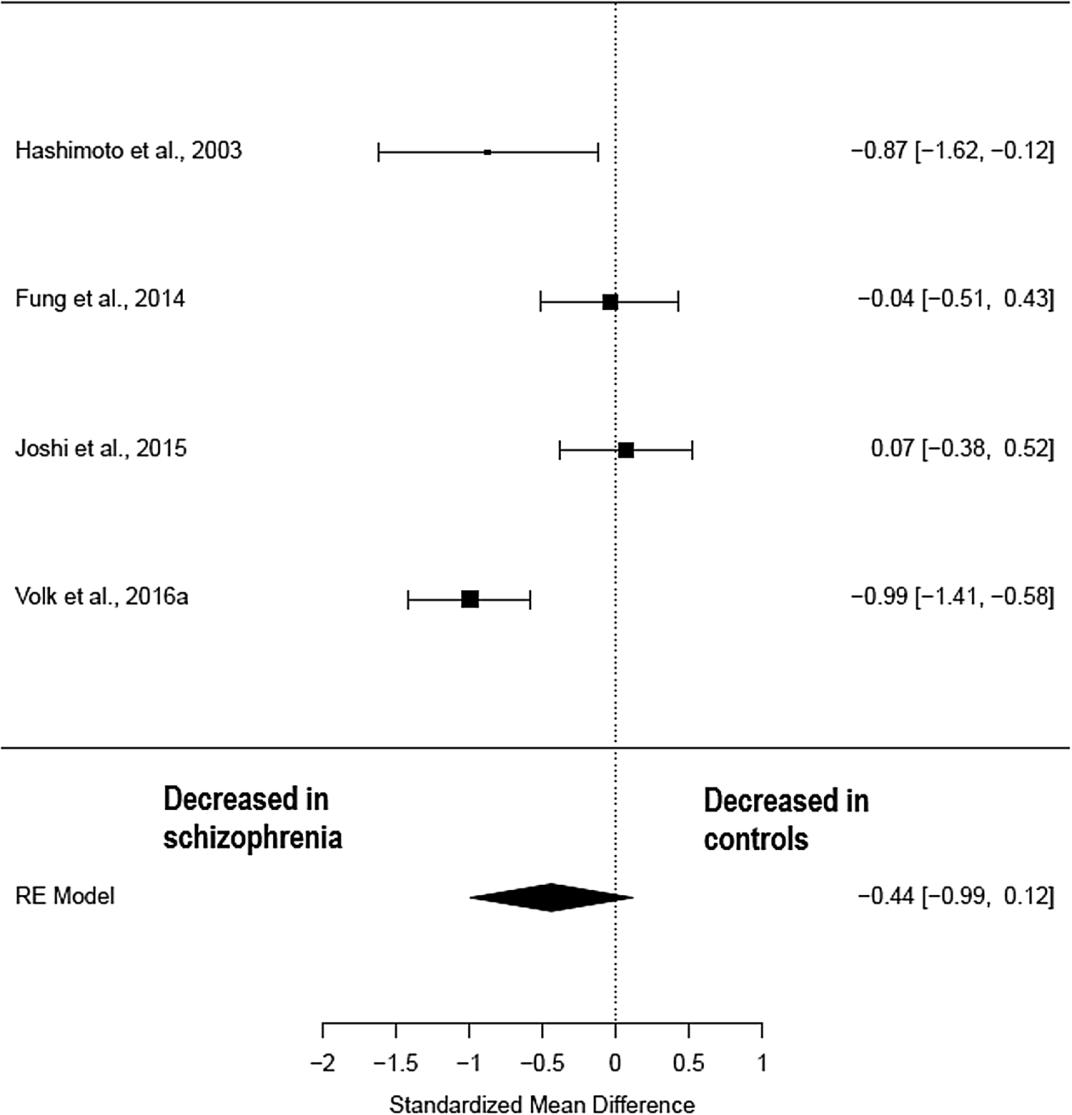
Meta-analysis of studies measuring parvalbumin mRNA in the pre-frontal cortex

Four studies (comprising 138 schizophrenia patients and 137 healthy controls) (Table 2) measured parvalbumin mRNA in pre-frontal regions. The meta-analysis showed a non-significant reduction in parvalbumin mRNA in patients with schizophrenia when compared to healthy controls (Hedges’ *g *= − 0.44; *z *= − 1.56; *p *= 0.12; 95% CI − 0.99–0.12) (Fig. 3). The *I*^2^ test revealed high heterogeneity suggesting significant differences between studies (*I*^2^ = 79.56%; 95% CI: 36.8–98.49%). Visual inspection of the funnel plot revealed one missing studies on the right-hand side and the regression was not significant (*t* = − 0.15; *df* = 2, *p* = 0.89) (Supplementary Fig. 1). Following correction for publication bias, the effect size decrease in parvalbumin mRNA in patients with schizophrenia remained unchanged (Hedges’ *g* = − 0.44; *p* = 0.12).

## Discussion

We found a significant reduction in parvalbumin cell density and a non-significant reduction in parvalbumin mRNA in patients with schizophrenia relative to healthy controls in pre-frontal cortical regions. Two studies of parvalbumin cell density in the frontal cortex showed large effect sizes, those by Hashimoto et al. (2003) and Bitanihirwe and Woo (2014); the latter uniquely used a cRNA GAT-1 probe and a DIG-labelled PV mRNA probe to measure the density of neurons expressing both parvalbumin and GABA transporter 1 (GAT-1) mRNA in cortical layers 3 and 4 (data included in this meta-analysis) in patients with schizophrenia. Therefore, this study measured a specific molecular subtype of parvalbumin interneuron and suggests that decreases in parvalbumin interneuron density in schizophrenia could be larger within discrete molecular classes of parvalbumin expressing interneurons. However, the study by Hashimoto et al. (2003) used a parvalbumin mRNA probe alone to identify parvalbumin-positive cells, and showed a greater effect size in this random-effects meta-analytic model, suggesting that reductions in parvalbumin cell density are likely to be generalised across different molecular sub-types of parvalbumin interneuron.

Our finding of a non-significant reduction in parvalbumin mRNA in frontal regions is unexpected, in particular given our finding of reduced parvalbumin cell density and the existence of micro-array studies which do show significant reductions in parvalbumin mRNA fontal regions (Enwright Iii et al. 2017; Mellios et al. 2009). Unfortunately, there were insufficient micro-array studies measuring parvalbumin expression in frontal regions to be included in a meta-analysis.

### Methodological considerations

**Fig. 4:**
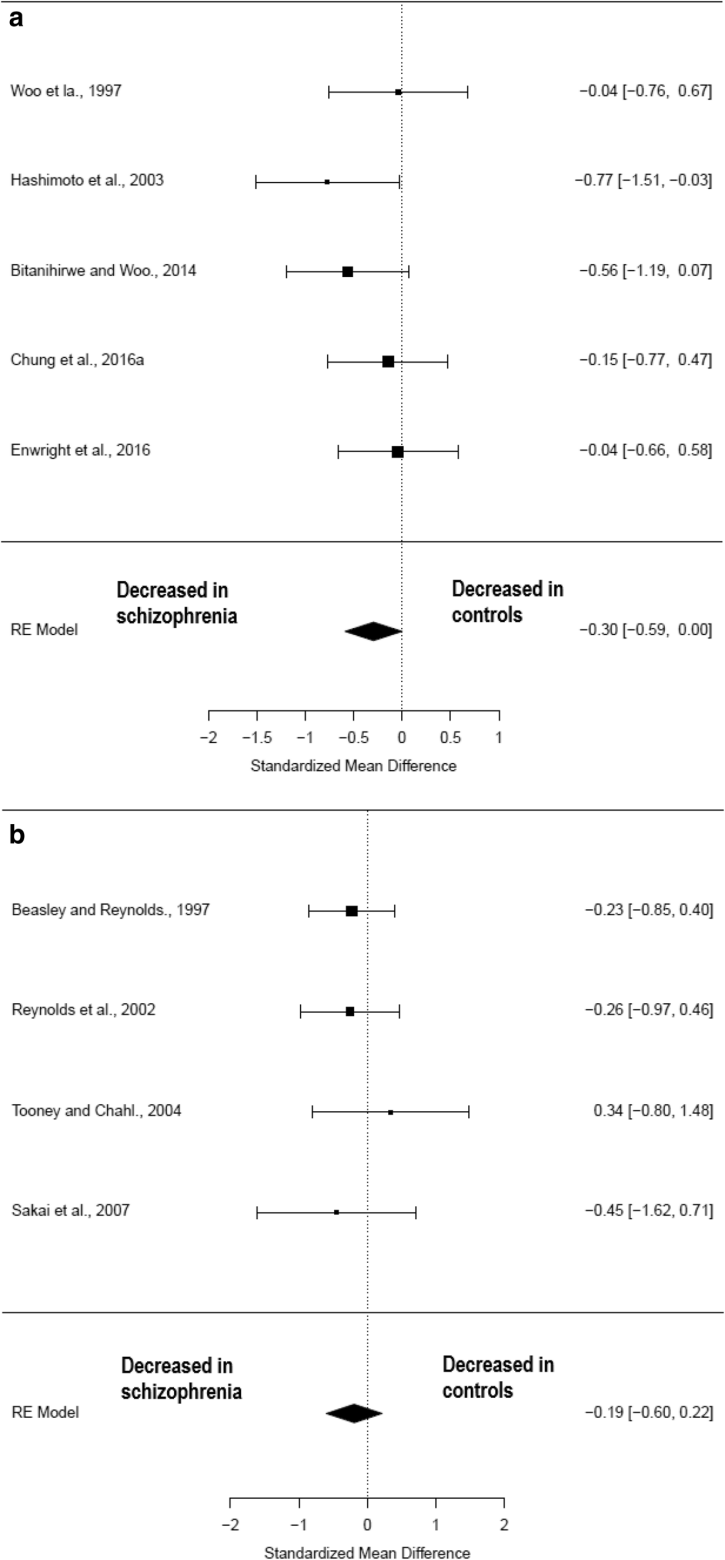
Sub-analysis of parvalbumin cell density in pre-frontal cortex by fixation method. **a** Paraformaldehyde (more reliable fixation method). **b** Paraffin/formalin (less likely to detect PV protein)

In terms of limitations, combining data from different studies in the context of meta-analyses lead to several potential sources of heterogeneity, such as methodological differences in labelling and microscopy techniques, and sample differences in illness duration, co-morbidity or prior treatment history of the patients included. All included studies took rigorous steps to ensure a reliable diagnosis of schizophrenia was confirmed according to established criteria, though several studies included patients with schizoaffective disorder as well as co-morbid substance misuse or dependence. Co-morbid alcohol dependence could be a significant confounding factor, in particular when looking at hippocampal pathology due to chronic alcohol-related changes in hippocampal size and volume (Agartz et al. 1999), though ultimately there were insufficient hippocampal studies to include in a quantitative meta-analysis. Co-morbidity within controls was also present in at least one study (Hashimoto et al. 2003). The method of tissue fixation, in particular the use of formalin and paraffin, may lead to a reduction in immunoreactive labelling of parvalbumin protein due to the effects of epitope masking, degradation and cross-linking (Ahram et al. 2003; Hoetelmans et al. 2001). Consistent with this, the sub-analysis by fixation method in the frontal cortex, suggests that effects were larger in the paraformaldehyde group than the formalin group, suggesting future studies should use paraformaldehyde fixation (see Fig. 4). Furthermore, Enwright et al. (2016) suggest that studies which use monoclonal antibodies for parvalbumin protein to measure parvalbumin cell density may overestimate the decrease in parvalbumin cell density due to low protein levels rendering some parvalbumin cells undetectable, except at much higher levels of magnification. Furthermore, it is likely that the stereotactic three-dimensional counting techniques used by later studies provide a more reliable counting method because cell size can be more accurately measured when calculating density. However, Sakai et al. (2008) noted that accurate estimates of cell density can be made using two-dimensional counting methods if the Abercrombie (Abercrombie and Johnson 1946) correction is used to adjust for the confounding effect of cell size.

**Fig. 5:**
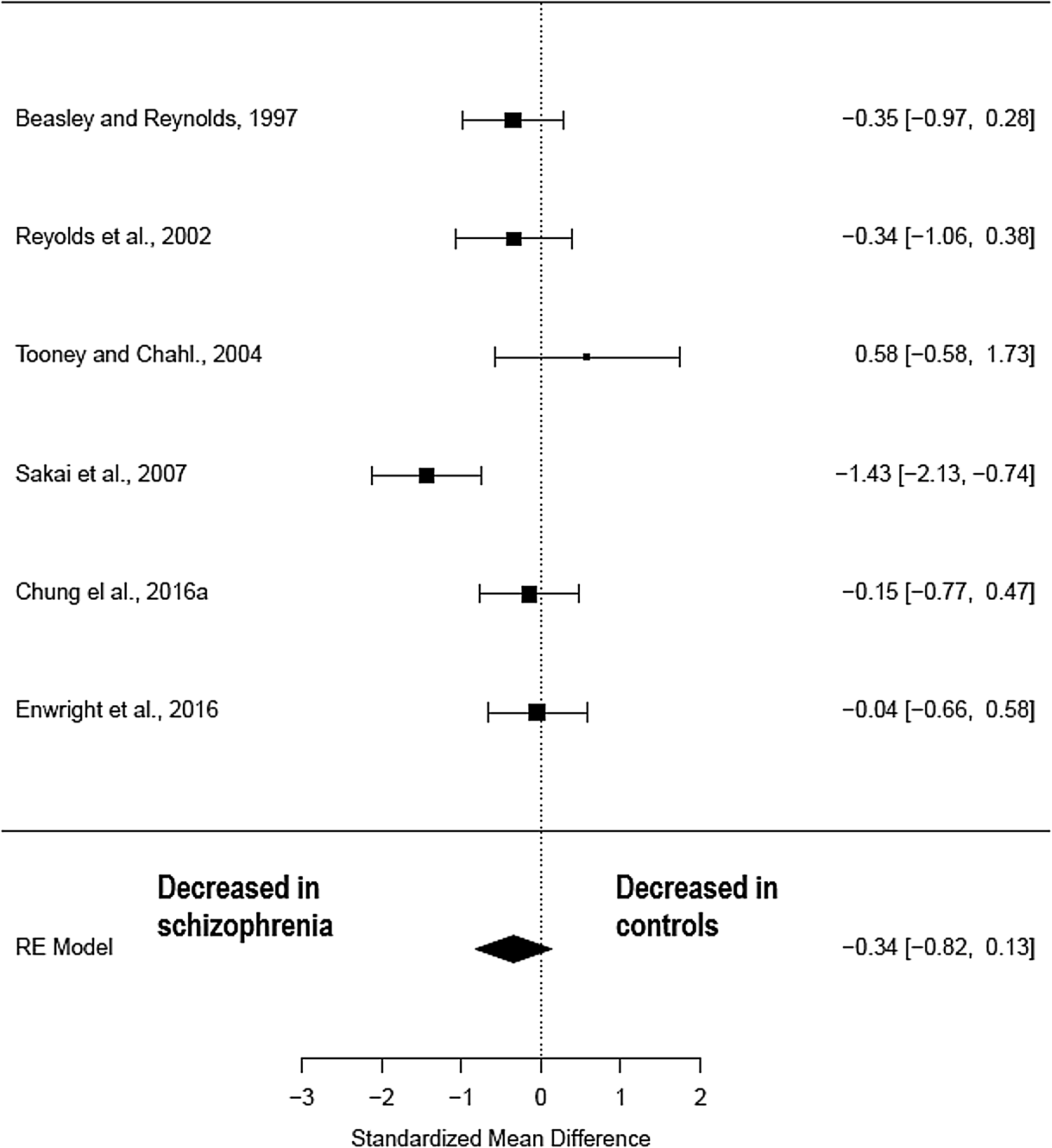
Sub-analysis of parvalbumin cell density in pre-frontal cortex layers 3 and 4 only

In our calculation of mean differences between patients and controls we averaged findings across grey matter layers for each region of the frontal cortex to obtain an overall mean and SD for that particular region. Parvalbumin interneurons are predominantly found in the middle and lower cortical layers (Hof et al. 1999; Tooney and Chahl 2004) and have been shown to be most affected in layers 3 and 4 in schizophrenia (Chung et al. 2016b; Hashimoto et al. 2003; Sakai et al. 2008; Tooney and Chahl 2004), and it maybe that averaging across layers underestimates the greater differences found between patients with schizophrenia and controls. We performed a sub-analysis for the studies that measured only layers 3 and 4 and found no significant difference between controls and patients for parvalbumin cell density in the frontal cortex (Hedges’ *g* = − 0.34 *z *= − 1.40 *p *= 0.16 95% CI: − 0.81–0.13—see Fig. 5).

Cause of death did differ between patients and controls with nearly all studies having an excess of suicides within the patient sample, except Sakai et al. (2008) which excluded cases of suicide. Cause of death is known to be a significant potential confounding factor in post-mortem studies as agonal factors such as hypoxia, head injury and ingestion of neurotoxic substances are known to have significant effects on RNA integrity in the post-mortem brain (Tomita et al. 2004). However, it is not known whether parvalbumin interneurons are susceptible to this effect. Brain pH, an inverse correlate with agonal state, was controlled for within each study and was not found to be significantly different between patients and controls. Post-mortem interval was significantly different between patients and controls in one early study Reynolds et al. (2002); however, the authors found no correlation between PMI and neuronal density. A meta-regression was performed with PMI as a co-variant in the studies measuring parvalbumin cell density and the relationship was not significant (*z *= − 0.05, *p *= 0.96) nor was year of publication (*z *=0.03, *p *= 0.97). However, in the meta-regression of parvalbumin mRNA studies post-mortem interval was significantly associated with the effect (Hedges’ *g* = 0.59, CI 0.29–0.89, *p* = <0.001). This suggests that the two studies which showed less of an effect in patients, Fung et al. (2014) and Joshi et al. (2015), may have done so because the difference in post-mortem interval was greater in patients versus controls. Again, year of publication was not significantly associated with the effect (*z* = 0.55, *p* = 0.58). It should also be noted that of the four studies included in the parvalbumin mRNA meta-analysis, the two which showed a difference in patients were from the Lewis lab, whereas the two which showed no difference were from the Weickert lab (see Fig. 3), so laboratory maybe a confounder for this meta-analysis.

Antipsychotic medication exposure in patients is a potential source of heterogeneity in findings, although antipsychotic treatment has not been associated with parvalbumin interneuron loss or changes in parvalbumin transcript levels in pre-clinical models (Hashimoto et al. 2003; Lewis et al. 2008).

A final methodological consideration is the effect of statistical test. Hashimoto et al. (2003) found a reduction in parvalbumin mRNA-positive neurons that was not significant using an analysis of covariance (ANCOVA) model. However, when the same mean and sd data were inputted into the meta-analysis, it was found to exert a significant effect. This suggests that when other confounding factors are taken into account, the effect size showing a reduction in patients is reduced.

### Implications for understanding the neurobiology of schizophrenia

Post-mortem studies of parvalbumin interneurons in schizophrenia initially focussed on interneuron morphology (Beasley and Reynolds 1997; Benes et al. 1991, 1998; Bernstein et al. 2007; Cotter et al. 2002; Danos et al. 1998; Falkai et al. 2016; Kalus et al. 1997; Konradi et al. 2011; Pantazopoulos et al. 2007, 2010; Reynolds et al. 2000; Wang et al. 2011; Woo et al. 1997; Zhang et al. 2002), while later studies began to measure parvalbumin-related proteins, mRNA and other molecular markers such as GAD_67_, GAT-1, ErB4q and PGC-1α (Bullock et al. 2008; Byne et al. 2008; Chung et al. 2016b; Fung et al. 2010; Hashimoto et al. 2003; Joshi et al. 2014; Lucas et al. 2014; McMeekin et al. 2016; Mellios et al. 2009). Our findings extend other evidence of GABAergic dysfunction, such as reduced levels of GAD_67_ in the frontal cortex (Fung et al. 2010; Volk et al. 2000), to identify a particular sub-class of GABAergic interneuron, parvalbumin-positive interneurons, as being affected in schizophrenia, although they do not exclude other sub-types also being involved.

Although our meta-analysis of parvalbumin mRNA in the pre-frontal cortex found a non-significant reduction, a recent large micro-array study of parvalbumin mRNA per parvalbumin-containing neuron in layer three of the dorsolateral pre-frontal cortex found a significant reduction of 22.2% in patients with schizophrenia (Enwright Iii et al. 2017), although it should be noted that this study contains sample overlap with a number of the group’s other studies in frontal regions (see “Materials and methods”), including Volk et al. (2016a) which is included in our PV mRNA meta-analysis. In addition, reduced interneuron PV mRNA appears to be associated with a concurrent loss of GAD_67_ mRNA (Chung et al. 2016a; Hashimoto et al. 2003), which could be due to defects within the PV gene (located 22q12–q13.1) or a factor that regulates gene expression. Toker et al. (2018) who used micro-array expression profiles of cell type marker genes in bulk post-mortem tissue as surrogate measures of cellular abundance suggest that their findings demonstrate the existence of a reduction in parvalbumin cell numbers in schizophrenia. Such findings suggest a deficiency secondary to either neuronal loss or altered parvalbumin transcription (Chung et al. 2016a; Glausier et al. 2014).

Two main morphological sub-types of parvalbumin interneurons have been characterised within the neocortex: fast-spiking chandelier or axo-axonic cells, which target the axon of pyramidal cells, and fast-spiking basket cells that synapse onto pyramidal cells in the perisomatic basket region (Cobb et al. 1995; Klausberger et al. 2003). Parvalbumin basket interneurons receive direct thalamic input (Jones 1993) and are thought to represent 80–90% of parvalbumin interneurons (Zaitsev et al. 2005). Such cells play a primary role in timing and synchronising pyramidal cell discharge through regulating rhythmic hyperpolarisation and local feedback loops (Bartos et al. 2007; Hu et al. 2014; Somogyi and Klausberger 2005; Tremblay et al. 2016). In doing so, they contribute to global and regional neuronal oscillations, within the gamma range (Gonzalez-Burgos et al. 2015; Senkowski and Gallinat 2015). Abnormalities in both gamma oscillation synchronicity and power are known to be present in schizophrenia (Gallinat et al. 2004; Kwon et al. 1999; Matthew et al. 2005; Spencer et al. 2004). Therefore, the reduction in parvalbumin interneurons we found in frontal regions in schizophrenia could contribute to the gamma power and synchrony alterations seen in the disorder and the disrupted cortical–thalamic connections which are thought to underlie cognitive dysfunction found in schizophrenia (Cho et al. 2006).

Our finding of reduced parvalbumin cell density in the pre-frontal cortex taken with other evidence implicating parvalbumin interneuron dysfunction (Enwright et al. 2016; Sohal et al. 2009; Volk et al. 2016b; Zhou et al. 2015), suggests that targeting parvalbumin interneurons may have potential as drug targets in the treatment of schizophrenia. One such possibility is agents that modulate Kv3.1/2 channels that are highly expressed on fast-spiking parvalbumin interneurons (Boddum et al. 2017; Brown et al. 2016; Rosato-Siri et al. 2015). Future post-mortem work on the molecular differentiation of parvalbumin interneuron subclasses and the regulational factors that act upon them will be needed to determine the true nature of parvalbumin interneuron pathology in schizophrenia.

## Conclusions

In summary, this meta-analysis finds that in schizophrenia parvalbumin interneurons in the frontal cortex show reduced neuronal density but the reduction in parvalbumin mRNA levels was not found to be significant. Both these findings should be viewed in the context of several methodological considerations and potential confounding factors. This highlights the need for further robust post-mortem studies in this and other brain regions, which take into account such factors. These findings add to the evidence that pathology of discrete neuronal classes exists in schizophrenia and that GABAergic interneurons play a significant role in the pathoetiology of the illness.

## Electronic supplementary material

Below is the link to the electronic supplementary material.

Supplementary material 1 (DOCX 117 kb)
