# Supplementary material for: Pre-frontal parvalbumin interneurons in schizophrenia: a meta-analysis of post-mortem studies
Source: J Neural Transm (Vienna). 2019 Sep 16;126(12):1637–51. doi: 10.1007/s00702-019-02080-2 (PMC6856257; doi:10.1007/s00702-019-02080-2)
Supplement: Supplementary file 1 — Supplementary material 1 (DOCX 117 kb) [file 702_2019_2080_MOESM1_ESM.docx]

**SUPLEMENTARY INFORMATION**

**Keyword and free text search**

1. exp Schizophrenia, Catatonic/ or exp Schizophrenia, Paranoid/ or exp Schizophrenia/ or exp Schizophrenia, Disorganized/ or exp Schizophrenia, Childhood/

2. exp Psychotic Disorders/

3. schizo*.mp. [mp=title, abstract, original title, name of substance word, subject heading word, keyword heading word, protocol supplementary concept word, rare disease supplementary concept word, unique identifier]

4. (psychotic* or psychosis*).mp. [mp=title, abstract, original title, name of substance word, subject heading word, keyword heading word, protocol supplementary concept word, rare disease supplementary concept word, unique identifier]

5. exp Parvalbumins/

6. parvalbumin*.mp. [mp=title, abstract, original title, name of substance word, subject heading word, keyword heading word, protocol supplementary concept word, rare disease supplementary concept word, unique identifier]

7. 1 or 3

8. 2 or 4

9. 5 or 6

10. 7 or 8

11. 9 and 10

**Supplementary Information Table 1. Demographic, clinical and methodological data from studies identified for qualitative synthesis.**

**Legend:**

**SCZ = schizophrenia CON = healthy controls PV = Parvalbumin PFC = Pre-frontal cortex BA = Brodmann Area**

**CPZ = Chlorpromazine equivalent NOS = Not specified**

**↓ = decreased in schizophrenia vs. controls ↑ = increased in schizophrenia vs. controls ↔ = no difference between schizophrenia and controls**

|  | **Author & Year** | **Outcome measure** | **Schizophrenia (SCZ) patients/controls (CON) (n)** | **Age mean (sd) (years): schizophrenia (SCZ)/ controls (CON)** | **Method** | **Area** | **Cause of death** | **Patients’ Medication** | **Mean (sd) post mortem interval (hr): schizophrenia (SCZ) patients/ controls (CON)** | **Significant findings (*p* < 0.05) (schizophrenia relative to controls) (summary)** | **Included in the quantative meta-analsysis** |
| --- | --- | --- | --- | --- | --- | --- | --- | --- | --- | --- | --- |
| 1 | Beasley and Reynolds., 1997 | Parvalbumin (PV) cell density (neurons per mm^2^) | 18/22 | 69.5 (± 3.7) / 62.7 (± 4.0) | Immunocytochemistry (ICC) | Prefrontal (PFC) (BA 10) | Schizophrenia (SCZ) - unknown Control (CON) - all sudden death | Unavailable | Unavailable | ↓ parvalbumin (PV) interneuron density in frontal cortex | Yes - PFC parvalbumin cell density |
| 2 | Kalus et al., 1997 | PV cell density (neurons/mm^2^) | 5/5 | 60.2 (±20.6) / 66.0 (±20.1) | ICC | Anterior Cingulate Cortex (BA 24c) | No suicides | All SCZ received neuroleptic medication, no controls did | 42.6 (17.2)/ 23.6 (14.3) | ↑PV interneuron density in layers Va and Vb ↔ PV interneuron density in layers II, III, IV in ACC | No - too few comparable studies |
| 3 | Woo et al., 1997 | Relative density (neurons/mm^2^), laminar distribution and somal size of PV interneurons | 15/15 | 53.6 (± 13)/53.9 (± 13.8) | ICC | Pre Frontal Cortex (BA 9 and 46) and primary visual cortex (BA 17) | SCZ - 5 suicide CON - 0 suicide | SCZ: Antipsychotics NOS CON: x1 antipsychotic | 11.7 (± 5.6)/11.3(± 5.4) | ↔ PV interneuron density in prefrontal cortex | Yes - PFC parvalbumin cell density |
| 4 | Danos et., 1998 | PV cell density (neurons/mm^2^) | 12/15 | 54 (± 9.3)/53.1 (± 8.9) | ICC | Thalamus | SCZ - 2 suicide CON - 0 suicide | All patients had antipsychotic medication | 35.3 (± 13.4)/29.6 (± 13.9) | ↓ PV interneuron density in corticothalamic projections | No - too few comparable studies |
| 5 | Cotter at al., 2002 | PV cell density (neurons/mm^2^) | 15/ 15 | 44.5 (± 13.1) / 48.1 (± 10.7) | ICC | Anterior cingulate cortex (BA 24) | No suicide | Lifetime flufenazine equivalent (mg) Minimum 0; Median 35,000; Maximum 200,000. 5 SCZ treated with antidepressants | 33.7 (± 14.6) / 23.7 (± 9.94) | ↔ PV interneuron density in ACC | No - too few comparable studies |
| 6 | Reynolds et al., 2002 | Total cortical PV cell density (neurons/mm^2^) (mm^2^) | 15/15 | 44.2 (Range 25–62) / 48.1 (Range 29–68) | ICC | Dorsolateral Prefrontal cortex (BA 46) and entorhinal cortex | SCZ - 4 suicide CON - 0 suicide | 14/15 patients received antipsychotics | 33.7 (Range 12–61)/23.7 (Range 8–42) | ↓PV neuron density in DLPFC and EC | Yes - PFC parvalbumin cell density |
| 7 | Zhang and Reynolds., 2002 | PV cell density (neurons/mm^2^) | 15/ 15 | 44.2 (Range 25–62) / 48.1 (Range 29–68) | ICC | Hippocampus | SCZ - 4 suicide CON - 0 suicide | 14/15 patients received antipsychotics | 3.7 (Range 12–61)/23.7 (Range 8–42) | ↓ PV interneuron density in hippocampus | No too few comparable studies |
| 8 | Hashimoto el al., 2003 | PV cell density (neurons/mm^2^) and PV mRNA per neuron | 15/15 | 43.0 (± 12) / 43.3 (± 14.6) | ICC and In situ (ISH) film radiography | Prefrontal cortical region (BA 9) | SCZ - 4 suicide CON - 0 suicide | 3 SCZ unmedicated (antipsychotic) at death | 16.9 (± 8.0)/ 17.0 (± 5.8) | ↔ PV interneuron density in PFC ↓ PV interneuron mRNA in PFC | Yes - PFC parvalbumin cell density and mRNA |
| 9 | Tooney and Chahl., 2004 | Relative densities of PV cells (neurons/mm^2^) | 6/6 | 44 (± 16)/ 43 (± 14) | ICC | Prefrontal cortical region (BA 9) | SCZ - 2 suicide CON - 3 suicide | All SCZ had taken various antipsychotic medications | 16.9 ((±9.5)/ 20.6 ((±7.0) | ↔ PV interneuron density in PFC | Yes - PFC parvalbumin cell density |
| 10 | Berstein et al., 2007 | PV cell density (neurons/mm^3^) | 15/15 | SCZ 51.4 (Range 43 - 62) | ICC | Mammillary bodies (MB) | SCZ - 3 suicide CON - 0 suicide | All patients received antipsychotic medication during the course of their disease | SCZ Range (hrs) 19 - 48 / CON 26 -48 | ↓ PV interneuron density in MB | No - too few comparable studies |
| 11 | Pantazopoulos et al., 2007 | Total neuronal number, numeric density(neurons/mm^3^), and somatal size of PV interneurons | 10/16 | 62.4 (± 17.8)/64.1 (± 13.1) | ICC | Entohinal cortex (EC) | SCZ - 1 suicide CON - 1 suicide | In SCZ. Estimated daily chlorpromazine equivalent dose 0 - 3550mg | 20.3 (± 6.8)/21.5 (± 5.6) | ↔ PV interneuron density in EC | No - too few comparable studies |
| 12 | Sakai et al., 2008 | Laminar density of PV cells (neurons/mm^2^) | 7/5 | 47.4 (± 7.63)/56.8 (± 5.81) | ICC | Pre-frontal cortex (PFC) (BA 9) | SCZ = 2 cardiorespiratory failure, 1 renal failure, 3 cancer, 1 thyroid crisis. CON = 3 cardiorespiratory failure, 2 liver failure | Not available | Not available | ↓ PV interneuron density in PFC layers 4 | Yes - PFC parvalbumin cell density |
| 13 | Byne, 2008 | PV mRNA expression | 14/ 16 | 78.79 (±8.59)/ 76.6 (±11.62) | qPCR | Thalamus | All-natural causes | Not available | 934 mins (516)/ 510 (399*) | ↔ PV mRNA expression | No - too few comparable studies |
| 14 | Bullok, 2008 | PV mRNA expression | 13/13 | 43.5 (±11)/ 40.9 (±8) | qPCR | Cerebellum | SCZ - 2 suicides, none in CON | All on antipsychotics at time of death | 12.1(5.1)/16.5 (5.4) | ↔ PV mRNA expression | No - too few comparable studies |
| 15 | Pantazopoulos et al., 2010 | Density of PV cells (neurons/mm^3)^ | 11/15 | 62.3 (± 16.8)/65.9 (± 12.0) | ICC | Amygdala | SCZ = 1 suicide non in controls | Not available | 20.7 (6.6)/21.2 (5.7) | ↔ PV interneuron density | No - too few comparable studies |
| 16 | Wang et al., 2011 | PV cell density (neurons/mm^3^) | 11/17 | 56.8 (± 14.8)/ 55.2 (± 16.6) | Nissl-stain and parvalbumin-immunoreactivity | Entorhinal cortex (EC) and Parahippocampus (Phip) | SCZ = Accident, PE, 6 Cardiopulmonary arrest, COPD, Pneumonia, Sepsis, Suicide. CON = 3 MIs, 6 Cardiopulmonary arrest, Suicide, Colon cancer, Breast cancer, Pnemonia, COPD, Heart failure. | Not available | 23.0 (± 6.3)/ 23.6 (± 6.8) | ↓ PV interneuron density in EC and Phip | No - too few comparable studies |
| 17 | Bitanihirwe and Woo., 2014 | Density of cells positive for both PV and GAT-1 (neurons/mm2) | 20/20 | 60.2 (± 16.7)/ 60.4 (± 17.3) | Dual ISH for parvalbumin and GABA transporter GAT-1 | Prefrontal cortex (BA 9) | SCZ = 2 Suicide | SCZ - 17 multiple psychotropics | 19.8 (± 0.28)/ 18.7 (± 5.5) | ↓ GAT-1+ PV interneuron density | Yes - PFC parvalbumin cell density |
| 18 | Fung et al., 2014 | Parvalbumin mRNA in total grey matter | 35/34 | 42.6 (19 - 59)/43.8 (31-60) | qPCR | Dorsolateral prefrontal cortex (BA 46) and lateral Orbitofrontal cortex | SCZ - 7 suicide | 85004.3 (100335) (Lifetime Fluphenazine mg equiv.) | 31.4 (15.4)/29.5 (13.0) | ↔ PV mRNA in DLPFC or Lateral OFC | Yes - PFC parvalbumin mRNA |
| 19 | Joshi et al., 2015 | PV mRNA expression in grey matter | 38/38 | 52.24 (±14.52)/52.55(±14.51) | qPCR | Orbital frontal cortex | Not availiable | SCZ: 4PAT; 22PT; 1AT; 7T; 1T&AT; 3NK | 28.21(±13.57)/26.43(±11.69) | ↓ PV mRNA in OFC (↔ using qPCR) | Yes - PFC parvalbumin mRNA |
| 20 | Chung et al., 2016a | Number of putative excitatory synapses per surface area of PV interneurons and PV cell density (neurons/identical volume) (mm^2^) | 20/20 | 45.2 (± 11.8)/ 46.3 (± 12.1) | ICC | Dorsolateral Prefrontal (DLPFC) (Layer 4) (BA 9) | SCZ - 8 Suicide | SCZ 19/20 Antipsychotics | 15.4 (± 6.3)/16.4 (± 5.5) | ↔ PV neuron density in layer 4 ↓PV protein levels in cell bodies ↓ density of VGlut1+/PSD95+ puncta on PV interneurons | Yes - PFC parvalbumin cell density |
| 21 | Enwright et al., 2016 | PV cell density (neurons/m3) | 20/20 | 45.8 (±9.5)/47 (±10.1) | ICC | Dorsolateral Prefrontal (DLPFC) (BA 9) | SCZ: 3 Suicide | SCZ 17/20 Antipsychotics | 13.2 (±7.7/)13.1 (±6.2) | ↔ densities of PV cells and of PNNs in DLPFC ↓ PV immunoreactivity in cell bodies and in individual PNNs around PV cells | Yes - PFC parvalbumin cell density |
| 22 | Falkai et al., 2016 | PV cell density (neurons/m3) | 10/10 | 55.1 (±7.7)/ 50.2 (±10.1) | ICC | Posterior hippocampus | SCZ: 3 suicide | SCZ: 9/10 on antipsychotics (1/10 unknown) | 42.0 (±17.2)/36.8 (±20.3) | ↔ PV neuron density ↑ in SCZ in hippocampus CA1 | No - too few comparable studies |
| 23 | McMeekin at al., 2016 | PV mRNA expression | 33/32 | 42(±9)/44(±7) | qPCR | Anterior Cingulate Cortex | SCZ = 4 suicide, 0 | SCZ antipsychotic exposure: 52 267±62 062mg | 33.7 (12–61)/23.7 (8–42) | ↓ PV mRNA in OFC (↔ using qPCR) | No – too few comparable studies |
| 24 | Volk et al., 2016b | PV mRNA levels in grey matter | 39/87 | 48.8(±12.9)/49.8 (±12.9) | qPCR | Prefrontal cortex (BA 9) | SCZ: 10 suicide | 89.7% SCZ on antipsychotics at time of death | 20.2 (±9.4)/19.3(±6.1) | ↓ PV mRNA in "Low LGM" group ↔ PV mRNA in "non-LGM" group | Yes - PFC parvalbumin mRNA |

**Supplementary figure 1:** Funnel plot of studies reporting parvalbumin cell density (a) and MRNA (b) in the pre-frontal cortex

**a:**

**
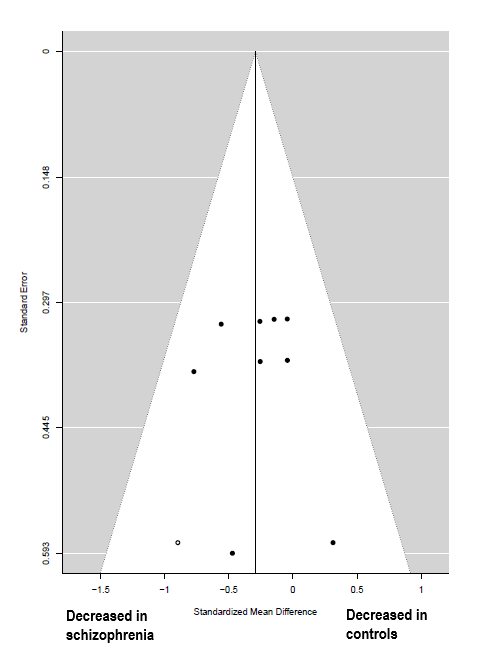
**

**b:**

**
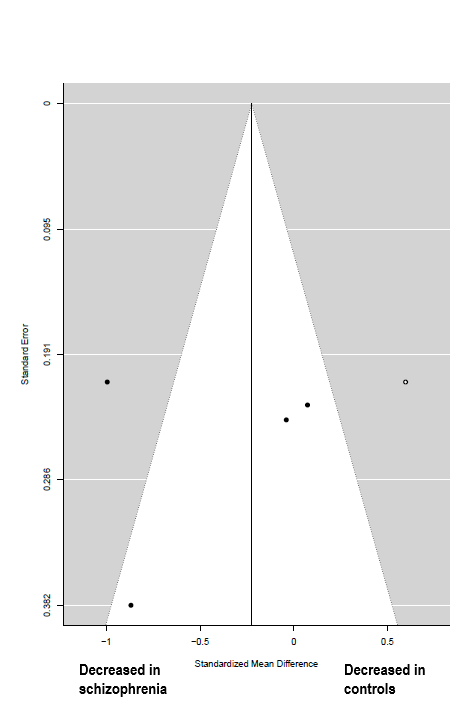
**

**Supplementary Figure 2.** Leave-one-out analysis for parvalbumin cell density studies


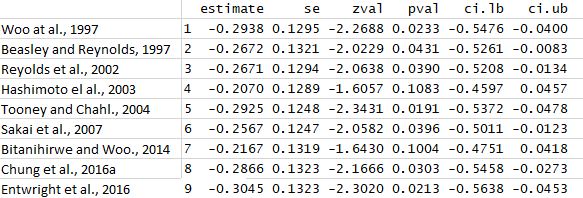


|  | **Full text list** |  |  |  |  |  |  |  |  |  |  |  |  |
| --- | --- | --- | --- | --- | --- | --- | --- | --- | --- | --- | --- | --- | --- |
|  | **Title** | **Authors** | **Journal and date of publication** | **Full text review** | **No scz group** | **Non-human** | **Not measuring PV density/mRNA** | **Review, abstract, book, not english etc** | **No data** | **Overlap** | **Micro-array** | **Includable** | **comments** |
| **1** | Human prefrontal layer ii interneurons in areas 46, 10 and 24. | Arteaga G. et al. | Colombia Medica. 46 (1) (pp 19-25), 2015. | 1 |  |  |  | 1 |  |  |  |  |  |
| **2** | Developmental patterns of prefrontal cortical perineuronal nets and their abnormalities in schizophrenia. | Athanas K. et al. | Schizophrenia Bulletin. Conference: 14th International Congress on Schizophrenia Research, ICOSR 2013. Publication: (var.pagings). 39 (SUPPL. 1) (pp S200), 2013. | 1 |  |  | 1 |  |  |  |  |  |  |
| **3** | Increased extracellular clusterin in the prefrontal cortex in schizophrenia. | Athanas et al. | Schizophrenia Research. 169 (1-3) (pp 381-385), 2015. | 1 |  |  | 1 |  |  |  |  |  |  |
| **4** | Comprehensive association analysis of 27 genes from the GABAergic system in Japanese individuals affected with schizophrenia. | Balan et al. | Schizophrenia Research. 185 (pp 33-40), 2017. | 1 |  |  | 1 |  |  |  |  |  |  |
| **5** | Heterogeneity of layer II neurons in human entorhinal cortex. | Beall MJ & Lewis DA. | Journal of Comparative Neurology. 321 (2) (pp 241-266), 1992. | 1 | 1 |  |  |  |  |  |  |  |  |
| **6** | Parvalbumin-immunoreactive neurons are reduced in the prefrontal cortex of schizophrenics. | Beasley, C & Reynolds, GP. | Schizophrenia Research. Vol.24(3), 1997, pp. 349-355. | 1 |  |  |  |  |  |  |  | 1 |  |
| **7** | Selective deficits in prefrontal cortical GABAergic neurons in schizophrenia defined by the presence of calcium-binding proteins. [References]. | Beasley, et al. | Biological Psychiatry. Vol.52(7), 2002, pp. 708-715. | 1 |  |  |  |  |  | 1 |  |  |  |
| **8** | Chronic adolescent exposure to delta-9-tetrahydrocannabinol in COMT mutant mice: impact on indices of dopaminergic, endocannabinoid and GABAergic pathways. | Behan et al. | Neuropsychopharmacology. 37(7):1773-83, 2012 Jun | 1 |  | 1 |  |  |  |  |  |  |  |
| **9** | Strongly reduced number of parvalbumin-immunoreactive projection neurons in the mammillary bodies in schizophrenia: Further evidence for limbic neuropathology. | Bernstein et al. | Signal Transduction Pathways, Part D: Inflammatory Signaling Pathways and Neuropathology. Annals of the New York Academy of Sciences. 1096 (pp 120-127), 2007. | 1 |  |  |  |  |  |  |  | 1 |  |
| **10** | Hippocampal expression of the calcium sensor protein visinin-like protein-1 in schizophrenia. | Bernstein et al. | Neuroreport. 13(4):393-6, 2002 Mar 25 | 1 |  |  | 1 |  |  |  |  |  |  |
| **11** | Perineuronal net abnormalities in schizophrenia. | Berretta et al. | Schizophrenia Bulletin. Conference: 14th International Congress on Schizophrenia Research, ICOSR 2013. Publication: (var.pagings). 39 (SUPPL. 1) (pp S201), 2013. | 1 |  |  |  | 1 |  |  |  |  |  |
| **12** | Perineuronal nets and schizophrenia: the importance of neuronal coatings. | Bitanihirwe BK & Woo TU. | Neuroscience and biobehavioral reviews. 45 (pp 85-99), 2014. | 1 |  |  |  | 1 |  |  |  |  |  |
| **13** | Glutamatergic deficits and parvalbumin-containing inhibitory neurons in the prefrontal cortex in schizophrenia. | Bitanihirwe et al. | BMC Psychiatry. 9 (no pagination), 2009. Article Number: 71. | 1 |  |  |  |  |  | 1 |  |  |  |
| **14** | Transcriptional dysregulation of gamma-aminobutyric acid transporter in parvalbumin-containing inhibitory neurons in the prefrontal cortex in schizophrenia. | Bitanihirwe BK &Woo TU. | Psychiatry Research. 220(3):1155-9, 2014 Dec 30 | 1 |  |  |  |  |  |  |  | 1 |  |
| **15** | A fundamental role for hippocampal parvalbumin in the dopamine hyperfunction associated with schizophrenia. | Boley et al. | Schizophrenia Research. 157(1-3):238-43, 2014 Aug | 1 |  | 1 |  |  |  |  |  |  |  |
| **16** | Altered expression of genes involved in GABAergic transmission and neuromodulation of granule cell activity in the cerebellum of schizophrenia patients. [References]. | Bullock et al. | The American Journal of Psychiatry. Vol.165(12), 2008, pp. 1594-1603. | 1 |  |  |  |  |  |  |  | 1 |  |
| **17** | Schizophrenia and sex associated differences in the expression of neuronal and oligodendrocyte-specific genes in individual thalamic nuclei | Byne et al. | Schizophr Res. 2008 Jan;98(1-3):118-28. Epub 2007 Oct 29. | 1 |  |  |  |  |  |  |  | 1 |  |
| **18** | A critical role for NMDA receptors in parvalbumin interneurons for gamma rhythm induction and behavior. | Carlen et al. | Molecular Psychiatry. 17(5):537-48, 2012 May | 1 |  | 1 |  |  |  |  |  |  |  |
| **19** | Role of ERBB4 splicing in parvalbumin interneuron maturation and schizophrenia. | Chung et al. | Neuropsychopharmacology. Conference: 56th Annual Meeting of the American College of Neuropsychopharmacology, ACNP 2017. United States. 43 (Supplement 1) (pp S317-S318), 2017. | 1 |  |  |  | 1 |  |  |  |  |  |
| **20** | Abnormal shift in ERBB4 splicing is associated with lower excitatory inputs onto parvalbumin-positive interneurons in subjects with schizophrenia. | Chung et al. | Schizophrenia Bulletin. Conference: 15th International Congress on Schizophrenia Research, ICOSR 2015. Conference Publication: (var.pagings). 41 (SUPPL. 1) (pp S248-S249), 2015. | 1 |  |  |  |  | 1 |  |  |  | overlap with chung a |
| **21** | Pathological basis for deficient excitatory drive to cortical parvalbumin interneurons in schizophrenia. | Chung et al. | American Journal of Psychiatry. 173 (11) (pp 1131-1139), 2016. | 1 |  |  |  |  |  |  |  | 1 | chung a |
| **22** | Shift in ERBB4 splicing is associated with developmental pruning of excitatory synapses on parvalbumin interneurons in monkey prefrontal cortex. | Chung at al. | Biological Psychiatry. Conference: 71st Annual Scientific Convention and Meeting of the Society of Biological Psychiatry, SOBP 2016. Conference Publication: (var.pagings). 79 (9 SUPPL. 1) (pp 325S), 2016. | 1 |  |  |  |  | 1 |  |  |  |  |
| **23** | Dysregulated ErbB4 splicing in schizophrenia: Selective effects on parvalbumin expression. | Chung et al. | American Journal of Psychiatry. 173 (1) (pp 60-68), 2016. | 1 |  |  |  |  |  | 1 |  |  | Chung b |
| **24** | The density and spatial distribution of GABAergic neurons, labelled using calcium binding proteins, in the anterior cingulate cortex in major depressive disorder, bipolar disorder, and schizophrenia. | Cotter et al. | Biological Psychiatry. 51(5):377-86, 2002 Mar 01 | 1 |  |  |  |  |  |  |  | 1 |  |
| **25** | A critical role for PGC-1alpha in the transcriptional control of parvalbumin-positive interneuron function. | Cowell R. | Neuropsychopharmacology. Conference: 50th Annual Meeting of the American College of Neuropsychopharmacology, ACNP.Conference Publication: (var.pagings). 36 (SUPPL. 1) (pp S71), 2011. | 1 |  |  | 1 |  |  |  |  |  |  |
| **26** | The expression of developmentally-regulated PGC-1alpha-dependent genes is reduced in the cortex of patients with schizophrenia. | Cowell et al. | Neuropsychopharmacology. Conference: 53rd Annual Meeting of the American College of Neuropsychopharmacology, ACNP 2014. Conference Publication: (var.pagings). 39 (SUPPL. 1) (pp S587), 2014. | 1 |  |  |  |  | 1 |  |  |  |  |
| **27** | Loss of association between PGC-1alpha and its putative targets in the dorsolateral prefrontal cortex of patients with schizophrenia. | Cowell et al. | Neuropsychopharmacology. Conference: 49th Annual Conference of the American College of Neuropsychopharmacology, ACNP 2010. Conference Publication: (var.pagings). 35 (SUPPL. 1) (pp S91), 2010. | 1 |  |  | 1 |  |  |  |  |  |  |
| **28** | A critical role for the transcription factor estrogen-related receptor-gamma in the regulation of gene expression and function of parvalbumin-positive neurons. | Cowell et al. | Neuropsychopharmacology. Conference: 54th Annual Meeting of the American College of Neuropsychopharmacology, ACNP 2015. Hollywood, FL United States. Conference Publication: (var.pagings). 40 (SUPPL. 1) (pp S560), 2015.. | 1 |  |  | 1 |  |  |  |  |  |  |
| **29** | Cortical deficits of glutamic acid decarboxylase 67 expression in schizophrenia: Clinical, protein, and cell type-specific features. | Curley et al. | American Journal of Psychiatry. 168 (9) (pp 921-929), 2011. | 1 |  |  | 1 |  |  |  |  |  |  |
| **30** | Role of glutamic acid decarboxylase 67 in regulating cortical parvalbumin and GABA membrane transporter 1 expression: implications for schizophrenia. | Curley et al. | Neurobiology of Disease. 50:179-86, 2013 Feb | 1 |  | 1 |  |  |  |  |  |  |  |
| **31** | Schizophrenia and anteroventral thalamic nucleus: Selective decrease of parvalbumin-immunoreactive thalamocortical projection neurons. | Danos et al. | Psychiatry Research: Neuroimaging. Vol.82(1), 1998, pp. 1-10. | 1 |  |  |  |  |  |  |  | 1 |  |
| **32** | Altered expression of ARP2/3 complex signaling pathway genes in prefrontal layer 3 pyramidal cells in schizophrenia. | Datta et al. | American Journal of Psychiatry. 174 (2) (pp 163-171), 2017. | 1 |  |  | 1 |  |  |  |  |  |  |
| **33** | Parvalbumin-immunoreactive neurons in the human anteroventral thalamic nucleus. | Dixon et al. | NeuroReport. 11 (1) (pp 97-101), 2000. | 1 | 1 |  |  |  |  |  |  |  |  |
| **34** | Transcriptome alterations of prefrontal cortical parvalbumin neurons in schizophrenia. | Enwright et al. | Molecular Psychiatry. 2017 Nov 07 | 1 |  |  |  |  |  | 1 |  | Microarray | overlap with chung, lewis, volk |
| **35** | Transcriptome profiling of layer 3 parvalbumin neurons from the dorsolateral prefrontal cortex of schizophrenia subjects. | Enwright et al. | Biological Psychiatry. Conference: 71st Annual Scientific Convention and Meeting of the Society of Biological Psychiatry, SOBP 2016. Conference Publication: (var.pagings). 79 (9 SUPPL. 1) (pp 236S), 2016. | 1 |  |  |  |  | 1 |  |  |  |  |
| **36** | Reduced labeling of parvalbumin neurons and perineuronal nets in the dorsolateral prefrontal cortex of subjects with schizophrenia. | Enwright et al. | Neuropsychopharmacology. 41 (9) (pp 2206-2214), 2016. | 1 |  |  |  |  |  |  |  | 1 |  |
| **37** | The ventral portion of the CA1 region of the hippocampus and the prefrontal cortex as candidate regions for neuromodulation in schizophrenia. | Ewing SG & Winter C. | Medical Hypotheses. 80(6):827-32, 2013 Jun | 1 |  |  |  | 1 |  |  |  |  |  |
| **38** | Oligodendrocyte and interneuron density in hippocampal subfields in schizophrenia and association of oligodendrocyte number with cognitive deficits. | Falkai et al. | Front Cell Neurosci. 2016 Mar 30;10:78. doi: 10.3389/fncel.2016.00078. eCollection 2016. | 1 |  |  |  |  |  |  |  | 1 |  |
| **39** | Decreased calretinin/glutamic acid decarboxylase 67 immunoreactive boutons in the prefrontal cortex of subjects with schizophrenia. | Fish et al. | Neuropsychopharmacology. Conference: 53rd Annual Meeting of the American College of Neuropsychopharmacology, ACNP 2014. Conference Publication: (var.pagings). 39 (SUPPL. 1) (pp S544-S545), 2014. | 1 |  |  | 1 |  |  |  |  |  |  |
| **40** | Differential expression of GAD67/GAD<inf>65</inf>-relevance to schizophrenia. | Fish KN. | Biological Psychiatry. Conference: 64th Annual Scientific Convention and Meeting of the Society of Biological Psychiatry. Conference Publication: (var.pagings). 65 (8 SUPPL. 1) (pp 15S), 2009. | 1 |  |  | 1 |  |  |  |  |  |  |
| **41** | Chandelier cell inputs to pyramidal neurons in schizophrenia and development. | Fish et al. | Neuropsychopharmacology. Conference: 50th Annual Meeting of the American College of Neuropsychopharmacology, ACNP. Conference Publication: (var.pagings). 36 (SUPPL. 1) (pp S219), 2011. | 1 |  |  | 1 |  |  |  |  |  |  |
| **42** | Alterations in density of gabaergic boutons in the prefrontal cortex in schizophrenia. | Fish at al. . | Biological Psychiatry. Conference: 69th Annual Scientific Convention and Meeting of the Society of Biological Psychiatry, SOBP 2014. Conference Publication: (var.pagings). 75 (9 SUPPL. 1) (pp 79S), 2014. | 1 |  |  | 1 |  |  |  |  |  |  |
| **43** | Schizophrenia and bipolar disorder show both common and distinct changes in cortical interneuron markers. | Fung et al. | Schizophrenia Research. 155 (1-3) (pp 26-30), 2014. | 1 |  |  |  |  |  |  |  | 1 |  |
| **44** | Expression of interneuron markers in the dorsolateral prefrontal cortex of the developing human and in schizophrenia. | Fung et al. | American Journal of Psychiatry. 167 (12) (pp 1479-1488), 2010. | 1 |  |  |  |  |  |  | 1 | Micro array |  |
| **45** | Reduced somatostatin and vasoactive intestinal peptide mRNAs in the frontal cortex of subjects with schizophrenia and bipolar disorder. | Fung SJ & Weickert CS | Neuropsychopharmacology. Conference: 52nd Annual Meeting of the American College of Neuropsychopharmacology, ACNP 2013. Conference Publication: (var.pagings). 38 (SUPPL. 2) (pp S317), 2013. | 1 |  |  | 1 |  |  |  |  |  |  |
| **46** | Expression of interneuron markers in the dorsolateral prefrontal cortex of the developing human and in schizophrenia. [References]. | Fung et al. | The American Journal of Psychiatry. Vol.167(12), 2010, pp. 1479-1488. | 1 |  |  | 1 |  |  |  |  |  |  |
| **47** | Lower gene expression for KCNS3 potassium channel subunit In parvalbumin-containing neurons In the prefrontal cortex in schizophrenia. | Georgiev et al. | American Journal of Psychiatry. 171 (1) (pp 62-71), 2014. | 1 |  |  | 1 |  |  |  |  |  |  |
| **48** | Selective expression of KCNS3 potassium channel alpha-subunit in parvalbumin-containing GABA neurons in the human prefrontal cortex. | Georgiev et al. | PLoS ONE. 7 (8) (no pagination), 2012. Article Number: e43904. | 1 |  |  | 1 |  |  |  |  |  |  |
| **49** | Identification of molecular markers for GABA neuron subsets in the human cerebral cortex. | Georgiev et | Neuroscience Research. Conference: 33rd Annual Meeting of the Japan Neuroscience Society, Neuro 2010. Conference Publication: (var.pagings). 68 (SUPPL. 1) (pp e84), 2010. | 1 |  |  | 1 |  |  |  |  |  |  |
| **50** | Cell type-specific transcriptional and ultrastructural analyses of oxidative phosphorylation in the prefrontal cortex of schizophrenia subjects. | Glausier et al. | Neuropsychopharmacology. Conference: 55th Annual Meeting of the American College of Neuropsychopharmacology, ACNP 2016. 41 (Supplement 1) (pp S311-S312), 2016. | 1 |  |  | 1 |  |  |  |  |  |  |
| **51** | GABA<inf>a</inf> alpha1 subunit mrna expression in pyramidal cells and interneurons in the dorsolateral prefrontal cortex of schizophrenia subjects. | Glausier et al. | Schizophrenia Bulletin. Conference: 13th International Congress on Schizophrenia Research, ICOSR. Conference Publication: (var.pagings). 37 (SUPPL. 1) (pp 183-184), 2011. | 1 |  |  | 1 |  |  |  |  |  |  |
| **52** | Pre-and postsynaptic markers of cortical parvalbumin basket cell terminals in schizophrenia. | Glausier et al. | Schizophrenia Bulletin. Conference: 14th International Congress on Schizophrenia Research, ICOSR 2013. Conference Publication: (var.pagings). 39 (SUPPL. 1) (pp S202), 2013. | 1 |  |  | 1 |  |  |  |  |  |  |
| **53** | Altered parvalbumin basket cell inputs in the dorsolateral prefrontal cortex of schizophrenia subjects. | Glausier et al. | Molecular Psychiatry. 19 (1) (pp 30-36), 2014. | 1 |  |  | 1 |  |  |  |  |  |  |
| **54** | Characterization of inhibitory and excitatory parvalbumin synapses in human and monkey prefrontal cortex. | Glausier et al. | Biological Psychiatry. Conference: 71st Annual Scientific Convention and Meeting of the Society of Biological Psychiatry, SOBP 2016. Conference Publication: (var.pagings). 79 (9 SUPPL. 1) (pp 346S), 2016. | 1 |  |  | 1 |  |  |  |  |  |  |
| **55** | Selective pyramidal cell reduction of GABA A receptor alpha1 subunit messenger RNA expression in schizophrenia. | Glausier JR & Lewis DA. | Neuropsychopharmacology. 36 (10) (pp 2103-2110), 2011. | 1 |  |  | 1 |  |  |  |  |  |  |
| **56** | Transcriptomic evidence for immaturity of the prefrontal cortex in patients with schizophrenia. | Hagihara et al. | Molecular Brain. 7 (1) (no pagination), 2014. Article Number: 41. | 1 |  |  | 1 |  |  |  |  |  |  |
| **57** | Conserved regional patterns of GABA-related transcript expression in the neocortex of subjects with schizophrenia. | Hashimoto et al. | American Journal of Psychiatry. 165 (4) (pp 479-489), 2008. | 1 |  |  |  |  |  | 1 |  |  |  |
| **58** | Relationship of brain-derived neurotrophic factor and its receptor TrkB to altered inhibitory prefrontal circuitry in schizophrenia. | Hashimoto et al. | Journal of Neuroscience. 25 (2) (pp 372-383), 2005. | 1 |  |  |  |  |  | 1 |  |  |  |
| **59** | Gene expression deficits in a subclass of GABA neurons in the prefrontal cortex of subjects with schizophrenia. | Hashimoto et al. | Journal of Neuroscience, 2003 | 1 |  |  |  |  |  |  |  | 1 |  |
| **60** | Decreased KCNS3 K<sup>+</sup> channel alpha-subunit gene expression in parvalbumin-containing GABA neurons in the prefrontal cortex of subjects with schizophrenia. | Hashimoto et al. | Neuroscience Research. Conference: 34th Annual Meeting of the Japan Neuroscience Society, Neuroscience 2011. Conference Publication: (var.pagings). 71 (SUPPL. 1) (pp e105-e106), 2011. | 1 |  |  | 1 |  |  |  |  |  |  |
| **61** | Layer 3 Excitatory and Inhibitory Circuitry in the Prefrontal Cortex: Developmental Trajectories and Alterations in Schizophrenia. | Hoftman et al. | Biological Psychiatry. 81 (10) (pp 862-873), 2017. | 1 |  |  |  | 1 |  |  |  |  |  |
| **62** | Relationship between somatostatin and death receptor expression in the orbital frontal cortex in schizophrenia: a postmortem brain mRNA study. | Joshi et al. | npj Schizophrenia volume1, Article number: 14004 (2015) |  |  |  |  |  |  |  |  | 1 |  |
| **63** | Altered distribution of parvalbumin-immunoreactive local circuit neurons in the anterior cingulate cortex of schizophrenic patients. | Kalus et al. | Psychiatry Research - Neuroimaging. 75 (1) (pp 49-59), 1997. | 1 |  |  |  |  |  |  |  | 1 |  |
| **64** | Inhibitory cartridge synapses in the anterior cingulate cortex of schizophrenics. | Kalus et al. | Journal of Neural Transmission. 106 (7-8) (pp 763-771), 1999. | 1 |  |  | 1 |  |  |  |  |  |  |
| **65** | Cell-type specific alterations of cortical interneurons in schizophrenic patients. | Kalus et al. | Neuroreport. 13(5):713-7, 2002 Apr 16 | 1 |  |  | 1 |  |  |  |  |  |  |
| **66** | Parvalbumin-positive neurons in the basal forebrain and cortical gamma oscillations. | Kim et al. | Asia-Pacific Psychiatry. Conference: 15th Pacific Rim College of Psychiatrists Scientific Meeting, PRCP 2012. Conference Publication: (var.pagings). 4 (SUPPL. 1) (pp 85-86), 2012. | 1 |  | 1 |  |  |  |  |  |  |  |
| **67** | Molecular abnormalities in the major psychiatric illnesses: Classification and Regression Tree (CRT) analysis of post-mortem prefrontal markers. | Knable et al. | Molecular Psychiatry. 7 (4) (pp 392-404), 2002. | 1 |  |  | 1 |  |  |  |  | measuring protein - no change |  |
| **68** | Molecular abnormalities of the hippocampus in severe psychiatric illness: Postmortem findings from the Stanley Neuropathology Consortium. | Knable et al. | Molecular Psychiatry. 9 (6) (pp 609-620), 2004. | 1 |  |  |  |  |  | 1 |  | hippocampus overlap of zhang and reynolds |  |
| **69** | Hippocampal interneurons are abnormal in schizophrenia. | Konradi et al. | Schizophrenia Research. 131 (1-3) (pp 165-173), 2011. | 1 |  |  | 1 |  |  |  |  |  |  |
| **70** | Decreased number of somatostatin and parvalbumin-positive hippocampal interneurons in schizophrenia. | Konradi et al. | Neuropsychopharmacology. Conference: 49th Annual Conference of the American College of Neuropsychopharmacology, ACNP 2010. Conference Publication: (var.pagings). 35 (SUPPL. 1) (pp S181-S182), 2010. | 1 |  |  |  |  | 1 |  |  |  |  |
| **71** | The cerebral cortex is damaged in chronic alcoholics. | Kril et al. | Neuroscience. 79(4):983-98, 1997 Aug | 1 | 1 |  |  |  |  |  |  |  |  |
| **72** | Microarray analysis of post-mortem hippocampus from matched cohorts of subjects with schizophrenia, bipolar disorder, and major depressive disorder. | Lanz et al. | Schizophrenia Bulletin. Conference: 13th International Congress on Schizophrenia Research, ICOSR. Conference Publication: (var.pagings). 37 (SUPPL. 1) (pp 192), 2011. | 1 |  |  |  |  | 1 |  |  |  |  |
| **73** | Lamina-specific deficits in parvalbumin-immunoreactive varicosities in the prefrontal cortex of subjects with schizophrenia: Evidence for fewer projections from the thalamus. | Lewis et al. | American Journal of Psychiatry. 158 (9) (pp 1411-1422), 2001. | 1 |  |  | 1 |  |  |  |  |  |  |
| **74** | GABAergic local circuit neurons and prefrontal cortical dysfunction in schizophrenia. | Lewis DA. | Brain Res Brain Res Rev. 2000 Mar;31(2-3):270-6. | 1 |  |  |  |  |  | 1 |  | overlap/replication of earlier lewis work |  |
| **75** | Cell and receptor type-specific alterations in markers of GABA neurotransmission in the prefrontal cortex of subjects with schizophrenia. | Lewis et al. | Neurotoxicity Research. 14 (2-3) (pp 237-248), 2008. | 1 |  |  |  | 1 |  |  |  |  |  |
| **76** | Cortical PGC-1?-dependent transcripts are reduced in postmortem tissue from patients with schizophrenia. | McMeekin et al. | Schizophrenia Bulletin. 42 (4) (pp 1009-1017), 2016. | 1 |  |  |  |  |  |  |  | 1 |  |
| **77** | Impaired GABAergic neurotransmission in schizophrenia underlies impairments in cortical gamma band oscillations. | McNally et al. | Current Psychiatry Reports. 15 (3) (no pagination), 2013. Article Number: 346. | 1 |  |  |  | 1 |  |  |  |  |  |
| **78** | Parvalbumin-immunoreactive axon terminals in macaque monkey and human prefrontal cortex: Laminar, regional, and target specificity of type I and type II synapses. | Melchitzky et al. | Journal of Comparative Neurology. 408 (1) (pp 11-22), 1999. | 1 |  |  | 1 |  |  |  |  |  |  |
| **79** | Molecular Determinants of Dysregulated GABAergic Gene Expression in the Prefrontal Cortex of Subjects with Schizophrenia. | Mellios et al. | Biological Psychiatry. 65 (12) (pp 1006-1014), 2009. | 1 |  |  |  |  |  |  | 1 | microarray pv mRNA |  |
| **80** | Morphometric characterization of synapses in the primate prefrontal cortex formed by afferents from the mediodorsal thalamic nucleus. | Negyessy L & Goldman-Rakic PS | Experimental Brain Research. 164 (2) (pp 148-154), 2005. | 1 |  | 1 |  |  |  |  |  |  |  |
| **81** | Extracellular matrix-glial abnormalities in the amygdala and entorhinal cortex of subjects diagnosed with schizophrenia. | Pantazopoulos et al. | JAMA, 2010 | 1 |  |  |  |  |  |  |  | 1 |  |
| **82** | Parvalbumin Neurons in the Entorhinal Cortex of Subjects Diagnosed With Bipolar Disorder or Schizophrenia. | Pantazopoulos et al. | Biological Psychiatry 2007 | 1 |  |  |  |  |  |  |  | 1 |  |
| **83** | Schizophrenia and major depression have distinct profiles of altered of gabaneuron density in the auditory cerebral cortex. | Pergolizzi et al. | Schizophrenia Bulletin [0586-7614] Pergolizzi yr:2011 vol:37 pg:185 |  |  |  |  |  | 1 |  |  |  |  |
| **84** | Microarray analysis of parvalbumin-containing inhibitory neurons in the superior temporal gyrus in schizophrenia. | Pietersen et al. | Schizophrenia Research. Conference: 2nd Schizophrenia International Research Society Conference, SIRS 2010. Conference Publication: (var.pagings). 117 (2-3) (pp 371-372), 2010. | 1 |  |  |  |  | 1 |  |  |  |  |
| **85** | A comparative perspective on minicolumns and inhibitory GABAergic interneurons in the neocortex. | Raghanti et al. | Frontiers in Neuroanatomy. (FEB), 2010. | 1 |  |  |  | 1 |  |  |  |  |  |
| **86** | Understanding the neurotransmitter pathology of schizophrenia: selective deficits of subtypes of cortical GABAergic neurons. | Reynolds et al. | J Neural Transm (Vienna). 2002 May;109(5-6):881-9. | 1 |  |  |  |  |  |  |  | 1 |  |
| **87** | Increased DNA methylation in the parvalbumin gene promoter in schizophrenia-relationship with symptoms and drug treatment. | Reynolds et al. | European Neuropsychopharmacology. Conference: 30th European College of Neuropsychopharmacology Congress, ECNP 2017. 27 (Supplement 4) (pp S597-S598), 2017. | 1 |  |  | 1 |  |  |  |  |  |  |
| **88** | Neurochemical correlates of cortical GABAergic deficits in schizophrenia: Selective losses of calcium binding protein immunoreactivity. | Reynolds et al. | Brain Research Bulletin. 55 (5) (pp 579-584), 2001. | 1 |  |  |  | 1 |  |  |  |  |  |
| **89** | Changes in density of calcium-binding-protein-immunoreactive GABAergic neurons in prefrontal cortex in schizophrenia and bipolar disorder. | Sakai et al. | Neuropathology. 28 (2) (pp 143-150), 2008. | 1 |  |  |  |  |  |  |  | 1 |  |
| **90** | Myelination of parvalbumin interneurons: A parsimonious locus of pathophysiological convergence in schizophrenia. | Stedehouder J &Kushner SA. | Molecular Psychiatry. 22 (1) (pp 4-12), 2017. | 1 |  |  |  | 1 |  |  |  |  |  |
| **91** | Neurons expressing calcium-binding proteins in the prefrontal cortex in schizophrenia. | Tooney, PA & Chahl, LA | Progress in Neuro-Psychopharmacology & Biological Psychiatry. 28(2):273-8, 2004 Mar | 1 |  |  |  |  |  |  |  | 1 |  |
| **92** | Neurochemical markers for schizophrenia, bipolar disorder, and major depression in postmortem brains. | Torrey et al. | Biological Psychiatry. 57 (3) (pp 252-260), 2005. | 1 |  |  |  |  |  | 1 |  | microarray using data from zhanga and reynold 2002 all hippocampus |  |
| **93** | Expression of GABA neuron markers across the cortical visuospatial working memory network in Schizophrenia. | Tsubomoto et al. | Biological Psychiatry. Conference: 72nd Annual Scientific Convention and Meeting of the Society of Biological Psychiatry, SOBP 2017. 81 (10 Supplement 1) (pp S69), 2017. | 1 |  |  |  |  |  |  |  | reduced PV mRNA visuospatial |  |
| **94** | Transcription factor deficiency in cortical parvalbumin neurons in schizophrenia. | Volk et al. | Biological Psychiatry. Conference: 67th Annual Scientific Convention and Meeting of the Society of Biological Psychiatry. Conference Publication: (var.pagings). 71 (8 SUPPL. 1) (pp 122S), 2012. | 1 |  |  | 1 |  |  |  |  |  |  |
| **95** | Altered expression of developmental regulators of parvalbumin and somatostatin neurons in the prefrontal cortex in schizophrenia. | Volk et al. | Schizophrenia Research. 177 (1-3) (pp 3-9), 2016. | 1 |  |  | 1 |  |  |  |  |  |  |
| **96** | Cortical inhibitory neuron disturbances in schizophrenia: Role of the ontogenetic transcription factor Lhx6. | Volk et al. | Schizophrenia Bulletin. 40 (5) (pp 1053-1061), 2014. | 1 |  |  | 1 |  |  |  |  |  |  |
| **97** | Contribution of deficits in the ontogenetic transcription factor Lhx6 to cortical inhibitory neuron dysfunction in schizophrenia. | Volk et al. | Biological Psychiatry. Conference: 69th Annual Scientific Convention and Meeting of the Society of Biological Psychiatry, SOBP 2014. Conference Publication: (var.pagings). 75 (9 SUPPL. 1) (pp 80S), 2014. | 1 |  |  | 1 |  |  |  |  |  |  |
| **98** | A cross-species investigation into the role of Lhx6 in cortical inhibitory circuitry disturbances in schizophrenia. | Volk et al. | Neuropsychopharmacology. Conference: 52nd Annual Meeting of the American College of Neuropsychopharmacology, ACNP 2013. Conference Publication: (var.pagings). 38 (SUPPL. 2) (pp S118-S119), 2013. | 1 |  |  | 1 |  |  |  |  |  |  |
| **99** | Deficits in transcriptional regulators of cortical parvalbumin neurons in schizophrenia. | Volk et al. | American Journal of Psychiatry. 169 (10) (pp 1082-1091), 2012. | 1 |  |  | 1 |  |  |  |  |  |  |
| **100** | Cortical GABA markers identify a molecular subtype of psychotic and bipolar disorders. | Volk et al. | Psychological medicine. 46 (12) (pp 2501-2512), 2016. | 1 |  |  |  |  |  |  |  | 1 | Volk b |
| **101** | Elevated transcript levels for viral restriction factors in cortical endothelial cells in schizophrenia. | Volk et al. | Neuropsychopharmacology. Conference: 51st Annual Meeting of the American College of Neuropsychopharmacology, ACNP 2012. Conference Publication: (var.pagings). 38 (SUPPL. 1) (pp S224-S225), 2012. | 1 |  |  | 1 |  |  |  |  |  |  |
| **102** | Bipolar disorder type 1 and schizophrenia are accompanied by decreased density of parvalbumin- and somatostatin-positive interneurons in the parahippocampal region. | Wang et al. | Acta Neuropathol. 2011 Nov;122(5):615-26. Epub 2011 Oct 4. | 1 |  |  |  |  |  |  |  | 1 |  |
| **103** | OTX2 expression in human prefrontal cortex development and in subjects with schizophrenia. | Woo et al. | Schizophrenia Research. Conference: 4th Biennial Schizophrenia International Research Conference. Conference Publication: (var.pagings). 153 (SUPPL. 1) (pp S382-S383), 2014. | 1 |  |  | 1 |  |  |  |  |  |  |
| **104** | Gene expression throughout human postnatal development in single cell populations in the prefrontal cortex. | Woo et al. | Schizophrenia Research. Conference: 4th Biennial Schizophrenia International Research Conference. Conference Publication: (var.pagings). 153 (SUPPL. 1) (pp S154), 2014. | 1 |  |  | 1 |  |  |  |  |  |  |
| **105** | Messenger rna and microrna expression profiling of pyramidal neurons, parvalbumin-immunoreactive neurons dopamine neurons and oligodendrocytes in schizophrenia and parkinson's disease. | Woo et al. | Schizophrenia Research. Conference: 4th Biennial Schizophrenia International Research Conference. Conference Publication: (var.pagings). 153 (SUPPL. 1) (pp S254-S255), 2014. | 1 |  |  |  |  | 1 |  |  |  |  |
| **106** | Cidar research cores: Genetic and neurobiological investigation of schizophrenia progression biomarkers. | Woo TU & Petryshen T. | Schizophrenia Bulletin. Conference: 13th International Congress on Schizophrenia Research, ICOSR. Colorado Springs, CO United States. Conference Publication: (var.pagings). 37 (SUPPL. 1) (pp 95), 2011. | 1 |  |  |  |  | 1 |  |  |  |  |
| **107** | Schizophrenia and the parvalbumin-containing class of cortical local circuit neurons. | Woo et al. | The American Journal of Psychiatry. Vol.154(7), 1997, pp. 1013-1015. | 1 |  |  |  |  |  |  |  | 1 |  |
| **108** | Alterations in cortical neuronal pentraxins and GAD67 in Schizophrenia. | Zaki et al. | Biological Psychiatry. Conference: 71st Annual Scientific Convention and Meeting of the Society of Biological Psychiatry, SOBP 2016. Conference Publication: (var.pagings). 79 (9 SUPPL. 1) (pp 66S-67S), 2016. | 1 |  |  | 1 |  |  |  |  |  |  |
| **109** | A selective decrease in the relative density of parvalbumin-immunoreactive neurons in the hippocampus in schizophrenia. | Zhang ZJ & Reynolds GP | Schizophrenia Research. 55 (1-2) (pp 1-10), 2002. | 1 |  |  |  |  |  |  |  | 1 |  |
| **110** | A reduction of nonpyramidal cells in sector CA2 of schizophrenics and manic depressives | Benes el al. | Biological Psychiatry | 1 |  |  | 1 |  |  |  |  |  |  |
| **111** | Gene expression analysis implicates a death receptor pathway in schizophrenia pathology | Catts, VS & Wickert, CS. | PLOS One April 2012 Volume 7 Issue 4 | 1 |  |  |  |  |  | 1 |  |  | pv data from fung 2010 and not reported |
